# Dermal Delivery of the High-Molecular-Weight Drug Tacrolimus by Means of Polyglycerol-Based Nanogels

**DOI:** 10.3390/pharmaceutics11080394

**Published:** 2019-08-05

**Authors:** Fiorenza Rancan, Hildburg Volkmann, Michael Giulbudagian, Fabian Schumacher, Jessica Isolde Stanko, Burkhard Kleuser, Ulrike Blume-Peytavi, Marcelo Calderón, Annika Vogt

**Affiliations:** 1 Clinical Research Center for Hair and Skin Science, Department of Dermatology and Allergy, Charité—Universitätsmedizin Berlin, Corporate Member of Freie Universität Berlin, Humboldt-Universität zu Berlin, and Berlin Institute of Health, 10117 Berlin, Germany; 2 Institut für Chemie und Biochemie, Freie Universität Berlin, 14195 Berlin, Germany; 3 Institute of Nutritional Science, Department of Nutritional Toxicology, University of Potsdam, 14558 Nuthetal, Germany; 4 Department of Molecular Biology, University of Duisburg-Essen, 45147 Essen, Germany; 5 POLYMAT and Applied Chemistry Department, Faculty of Chemistry, University of the Basque Country UPV/EHU, Paseo Manuel de Lardizabal 3, 20018 Donostia-San Sebastián, Spain; 6 IKERBASQUE, Basque Foundation for Science, 48013 Bilbao, Spain

**Keywords:** tacrolimus formulation, nanogels, skin penetration, drug delivery, human excised skin, Jurkat cells

## Abstract

Polyglycerol-based thermoresponsive nanogels (tNGs) have been shown to have excellent skin hydration properties and to be valuable delivery systems for sustained release of drugs into skin. In this study, we compared the skin penetration of tacrolimus formulated in tNGs with a commercial 0.1% tacrolimus ointment. The penetration of the drug was investigated in ex vivo abdominal and breast skin, while different methods for skin barrier disruption were investigated to improve skin permeability or simulate inflammatory conditions with compromised skin barrier. The amount of penetrated tacrolimus was measured in skin extracts by liquid chromatography tandem-mass spectrometry (LC-MS/MS), whereas the inflammatory markers IL-6 and IL-8 were detected by enzyme-linked immunosorbent assay (ELISA). Higher amounts of tacrolimus penetrated in breast as compared to abdominal skin or in barrier-disrupted as compared to intact skin, confirming that the stratum corneum is the main barrier for tacrolimus skin penetration. The anti-proliferative effect of the penetrated drug was measured in skin tissue/Jurkat cells co-cultures. Interestingly, tNGs exhibited similar anti-proliferative effects as the 0.1% tacrolimus ointment. We conclude that polyglycerol-based nanogels represent an interesting alternative to paraffin-based formulations for the treatment of inflammatory skin conditions.

## 1. Introduction

Tacrolimus, also known as FK 506, is an immunosuppressive drug acting predominantly via inhibition of T cell proliferation. This macrolide molecule builds a complex with the FK binding protein, which in turn binds to calcineurin and inhibits the dephosphorylation of the nuclear factor of activated T cells (NFATs) and its translocation to the nucleus [1,2,3]. As a consequence, the expression of IL-2, which is required for T cell proliferation, is inhibited. Tacrolimus also binds to isoforms of the FK binding protein, which have a cell type-specific expression pattern. This results in inhibitory or toxic effects towards other types of cells, e.g., mast and Langerhans cells [4,5,6]. In addition, induction of T cell apoptosis was found by Hashimoto and co-workers [7] and confirmed by Chung et al., who showed that tacrolimus induced apoptosis in Jurkat cells via activation of caspases 3 and 12 [8]. Furthermore, interference with the activation of the mitogen-activated protein kinase (MAPK) signaling pathways in primary human T lymphocytes was described by Matsuda et al. [9].

While the compound is widely used as topical agent to treat certain inflammatory skin diseases, its limited penetration across the skin barrier is a major limitation for a wider clinical use. Because of the poor solubility, the marketed formulation (Protopic^®^) consists of a paraffin-based ointment containing 0.03% or 0.1% tacrolimus in hard, liquid and white soft paraffin. In addition to insufficient drug penetration rates, mis-sensations such as skin stinging, burning and pruritus at the application site frequently lead to treatment discontinuation [10]. These side effects probably result from drug-induced chemical irritation and release of pro-inflammatory cytokines such as IL-6, as demonstrated for UVB-activated keratinocytes [11] and fibroblasts [12].

The hydrophobic paraffins in the ointment can reduce skin water loss and have therefore moisturizing properties. However, besides the fact that greasy excipients may be unpleasant for daily skin care, increasing concerns have been expressed because of symptom exacerbation in eczema sufferers. Furthermore, paraffin is a flammable excipient and consumers are often not aware of this fact [13]. Thus, several aspects indicate that new alternative formulations could result in significant improvements of current therapeutic strategies. Consequently, new formulations for the delivery of tacrolimus into the skin have been investigated in the last years, including lipid-based nanocarriers [14], liposomes [15], micelles [16,17,18], or transferosomes [19]. In all these works, the investigated nanocarrier formulation improved skin penetration of tacrolimus with respect to the marketed formulation.

Almost all studies used lipid-based carriers or delivery systems formulated with surfactants or permeabilizing components like ethanol that may cause side effects when applied on inflamed skin. In contrast, in this study, we investigated an alternative formulations based on thermoresponsive nanogels (tNGs) made of dendritic polyglycerol, which is cross-linked with thermoresponsive polymers, namely poly(glycidyl methyl ether-co-ethyl glycidyl ether) (tPG) and poly(*N*-isopropylacrylamide) (pNIPAM). During inflammatory skin diseases, skin temperature is slightly enhanced compared to unaffected skin. The concept of thermoresponsive nanogels implies sensing of such temperature differences with preferential release of drug in diseased skin. In fact, the progressive temperature increment after topical application has been shown to influence nanogel softness and the release of the drug [20]. More specifically, nanogels are soft when applied on skin surface and can penetrate deep in the stratum corneum (SC), where local temperature increase induces a change of conformation that results in the release of the incorporated active compound. The investigated nanogels were shown to enhance SC hydration [21,22,23] and exhibited promising drug delivery properties [24] along with low cytotoxicity [25,26].

In most previous studies, tacrolimus penetration was investigated using animal skin [18,27], which is anatomically and immunologically different from human skin, with little consideration of skin barrier impairment or skin inflammatory status. In this study, we used excised human skin from two distinct anatomical regions, breast as well as abdomen, and different methods to induce barrier disruption and inflammatory reactions. In this way, we could monitor the effects of barrier-disruption on tacrolimus skin penetration and release of inflammatory markers like IL-6 and IL-8. The additional use of a trans-well set up to co-culture ex vivo human skin and Jurkat cells enabled the assessment of tacrolimus anti-proliferative effects.

## 2. Materials and Methods

### 2.1. Nanogel Preparation and Characterization

Commercially available chemicals from standardized sources were used as delivered. Solvents were purchased as reagent grade and distilled if necessary. Anhydrous solvents were either purchased as ultra-dry solvent (Acros Organics^®^, Geel, Belgium) or received from solvent purification system. For the tPG polymerization reactions, dry toluene was obtained from MBRAUN SPS 800 solvent purification system (Garching, Germany). Water was purified by Millipore water purification system. Dendritic polyglycerol with average molecular weight of 10 kDa (PDI = 1.27) was purchased from Nanopartica GmbH (Berlin, Germany). Glycidyl methyl ether (85%) and ethyl glycidyl ether (98%; both TCI Europe, Eschborn, Germany) were dried over CaH_2_, distilled, and stored over molecular sieves (5 Å). The crosslinking reagent (1R,8S,9s)-bicyclo[6.1.0]non-4-yn-9-ylmethyl (4-nitrophenyl) carbonate was purchased from Synaffix (Oss, the Netherlands). The cyanine dye indodicarbocyanine (IDCC) was purchased from Lumiprobe GmbH (Hannover, Germany).

The tNGs were synthesized according to previously reported methods. For detailed synthesis and characterization description, refer to the following publications [22,26,28]. Briefly, for the synthesis of poly(*N*-isopropylacrylamide) (pNIPAM)-based nanogels (NG-pNIPAM), NIPAM (66 mg), acrylated dendritic polyglycerol (dPG-Ac_10%_) (33 mg), sodium dodecyl sulfate (SDS) (1.8 mg), and ammonium persulfate (APS) (2.8 mg) were dissolved in 5 mL of distilled water. Argon was bubbled into the reaction mixture for 15 min, which was followed by stirring under argon atmosphere for another 15 min. The reaction mixture was transferred into a hot bath at 68 °C and polymerization was activated after 5 min with the addition of a catalytic amount of *N*,*N*,*N*′,*N*′-tetramethylethane-1,2-diamine (TEMED; 120 µL). The mixture was stirred at 500 rpm for at least 4 h, prior to purification by dialysis. For the synthesis of fluorescently labeled nanogels, a mixture of unlabeled dPG-Ac_10%_ (30 mg) and indodicarbocyanine (IDCC)-labelled dPG-Ac_10%_ (3 mg) was used.

For the synthesis of tPG-based nanogels (NG-tPG), dPG functionalized with (1R,8S,9s)-bicyclo[6.1.0]non-4-yn-9-ylmethyl carbonate (dPG-BCN_8%_) (10 mg) and di-azide functionalized tPG (tPG-(N_3_)_2_) (20 mg) were mixed in 1 mL of dimethylformamide (DMF), cooled in an ice bath and injected with a syringe into 20 mL of water at 45 °C. The mixture was stirred for 3 h and the unreacted alkynes were quenched with azidopropanol or alternatively with IDCC-N_3_.

For loading tacrolimus, highly concentrated tNGs (10 mg) were added to a suspension of tacrolimus (5 mg) in 2 mL of MilliQ water. The nanogels were let to swell, followed by sonication in an ice water bath for 30 min. The suspension was stirred overnight at 25 °C and the encapsulated fraction was separated from the free drug by filtration with a 0.45 µm regenerated cellulose syringe filter. Following, 100 µL of the encapsulated fraction were lyophilized for determination of the total concentration, while the encapsulation capacity was determined by LC-MS/MS measurements. The nanogels with the encapsulated drugs were stored at 4 °C. The characterization of the investigated nanogels is reported in Table 1.

### 2.2. Skin Samples

Breast and abdominal skin were obtained after informed consent from healthy donors undergoing plastic surgery. The study was conducted after approval by the Ethics Committee of the Charité–Universitätsmedizin Berlin (approval EA1/135/06, renewed on January 2018) and in accordance with the Declaration of Helsinki guidelines. The excised skin was used between 3 and 6 h after surgery and was examined to exclude injured parts, including macroscopic skin surface damages but also deeper structural defects like stretch marks. Subcutaneous fat tissue was partially removed (approximately 0.5 cm was kept) and skin was stretched and fixed on a Styrofoam block using needles. Skin areas of 2 cm^2^ were marked. In order to induce skin barrier disruption, three methods were used: tape stripping 50 times (50 × TS), laser poration (LP), and sodium lauryl sulfate (SLS). TS was performed with adhesive, polyethylene tape (19-mm diameter, TESA film no. 5529, Beiersdorf, Hamburg, Germany). For LP, a P.L.E.A.S.E^®^ Professional system (Pantec Biosolutions, Ruggell, Lichtenstein) was used with the following setting: delivered energy 73.1 J/cm^2^, pulse length 125 μs, repetition rate 300 Hz, 10 pulses per pore, treated surface 10 × 10 mm, pore density 8%. For SLS treatment, 20 µL/cm^2^ of 5 % SLS (*w*/*v*) in deionized distilled water were applied on a filter paper disc (12 mm^2^ diameter for Finn Chambers^®^, SmartPractice, Hillerød, Denmark) and incubated for 4 h at 37 °C. Thereafter, skin was carefully cleaned with a paper towel.

A total amount of 5 µg tacrolimus per square centimeter was applied either incorporated in an aqueous suspension of nanogels or as 0.1% marketed formulation (Protopic^®^, manufactured by LEO Laboratories Ltd., Dublin, Ireland). Safety margins of at least 0.5 cm were left. Controls consisted of skin treated with 20 µL/cm^2^ of sterile 0.9% NaCl solution. Samples and controls were placed in humid chambers (plastic boxes with lid filled with humidified towels) and incubated for 24 h at 37 °C, 5% CO_2_ and 100% humidity. After incubation, non-penetrated material was removed with a paper towel, the untreated safety margins were cut and the treated tissue blocks were plunge frozen in liquid nitrogen and stored at −80°C for further processing.

### 2.3. Preparation of Skin Extracts

In order to separate epidermis from dermis and prepare extracts for tacrolimus and cytokine analyses, skin samples were cut horizontally (thickness 50 µm) with a microtome (Frigocut 2800 N, Leica, Bensheim, Germany): the first 100 µm corresponding roughly to epidermis and the remaining 900 µm to dermis. The sections were put in 500 µL of extraction buffer (100 mM Tris-HCl; 150 mM NaCl; 1 mM EDTA; 1 g Triton-X-100; 10% EtOH), homogenized (GLH OMNI homogenizer, Kennesaw, GA, USA) for 10 s, and incubated on ice for 45 min. Then, samples were sonicated at 4 °C for 10 min, vortexed and centrifuged for 5 min at 450× *g*. The pellets were then added of 500 µL of extraction buffer and the above described procedure was repeated once more. The supernatants were pooled and stored at −80°C.

### 2.4. Determination of Tacrolimus in Skin Extracts by Isotope-Dilution Liquid Chromatography Tandem-Mass Spectrometry (LC-MS/MS)

Skin extracts were thawed at room temperature, spiked with stable-isotope labeled internal standard [^13^C_1_,D_4_]tacrolimus (Alsachim, Illkirch-Graffenstaden, France), vortexed, and centrifuged at 9300× *g* for 3 min (4 °C). Analyses were conducted with an Agilent 1260 Infinity LC system coupled to an Agilent 6490 triple quadrupole-mass spectrometer (both from Waldbronn, Germany) interfaced with an electrospray ion source operating in the positive ion mode (ESI+). Chromatographic separation was carried out using an Agilent Zorbax SB-C18 column (1.8 μm, 2.1 × 50 mm). Aqueous ammonium formate (20 mM, pH 3.5) and methanol (VWR, Darmstadt, Germany) were used as eluents A and B, respectively. Samples (5 µL) were injected into a mobile phase consisting of 90% eluent A. Tacrolimus and its internal standard [^13^C_1_,D_4_]tacrolimus were eluted from the column, which was tempered at 30 °C, with a 4-min linear gradient to and a subsequent isocratic stage for 5 min at 2:98 (*v*:*v*) eluent A/B at a flow rate of 0.35 mL/min. Tacrolimus and [^13^C_1_,D_4_]tacrolimus co-eluted from the separation column at 5.3 min. The total run time for one analysis was 13 min, including re-equilibration of the LC system. The following ion source parameters were taken from Koster et al. [29], who used a comparable mass spectrometric configuration for detection of four immunosuppressants including tacrolimus: drying gas temperature = 200 °C, drying gas flow = 13 L/min of nitrogen, sheath gas temperature = 200 °C, sheath gas flow = 12 L/min of nitrogen, nebulizer pressure = 18 psi, capillary voltage = 4500 V, and nozzle voltage = 0 V. The ion funnel parameters were: high pressure RF voltage = 150 V and low pressure RF voltage = 60 V. Quantification of tacrolimus in relation to the internal standard [^13^C_1_,D_4_]tacrolimus was carried out using the multiple reaction monitoring (MRM) approach. Ammonium adducts [M+NH_4_]^+^ were selected as precursor ions by the first quadrupole. The following mass transitions were recorded (optimized collision energies in parentheses): tacrolimus: *m*/*z* 821.5 → 786.5 (16 eV), *m*/*z* 821.5 → 768.5 (20 eV), *m*/*z* 821.5 → 576.2 (24 eV); [^13^C_1_,D_4_]tacrolimus: *m*/*z* 826.5 → 791.5 (16 eV), *m*/*z* 826.5 → 773.6 (20 eV), *m*/*z* 826.5 → 581.4 (24 eV). Thereby, the loss of two hydroxyl groups and ammonium from the precursor ion, represented by *m*/*z* 821.5 → 768.5 for tacrolimus and *m*/*z* 826.5 → 773.6 for [^13^C_1_,D_4_]tacrolimus, was used for quantification. The dwell time for each of the six mass transitions recorded was 150 ms.

### 2.5. Enzyme-linked Immunosorbent Assay (ELISA)

Human IL-6 and IL-8 were investigated using ELISA kits (Human IL-6 and IL-8 CytoSetTM (CHC1263, CHC1303) Invitrogen Corporation, Carlsbad, CA, USA) following the manufacturer instructions. The amounts of cytokines were normalized to total protein content measured with Pierce 660 nm Protein Assay (Thermo Fisher Scientific Inc., Rockford, IL, USA). Absorbance was measured with EnSpire^®^ Multimode plate reader (Perkin Elmer, Akron, OH, USA).

### 2.6. Preparation of Cryosections and Fluorescence Microscopy

Skin samples treated with fluorescent nanogels were placed in tissue freezing medium (Leica Microsystems, Wetzlar, Germany) and plunge-frozen in liquid nitrogen. Cryosections of 5 μm thickness were prepared and observed by means of a fluorescence microscope (Olympus BX60F3, Olympus, Hamburg, Germany). The following filter combinations were used: bright pass = 545–580 nm, long pass > 610 nm for IDCC (red) and bright pass = 470–490 nm, long pass > 550 nm for FL. Pictures (magnification of 200×) of at least 20 randomly chosen skin sections per donor and skin sample were taken, and the mean fluorescence intensity of areas in the SC, viable epidermis, and dermis was calculated using the ImageJ software (Version 1.47).

### 2.7. Isolation of Cells, Flow Cytometry, and Confocal Fluorescence Microscopy

After incubation with nanogels, skin samples were cut in small pieces (0.2 × 0.2 cm) and incubated overnight at 4 °C in 2.4 U/mL dispase (Roche Applied Science, Penzberg, Germany) in order to detach epidermis from dermis. The epidermis sheets were incubated for 10 min at 37 °C in 5 mL of trypsin solution (0.025% trypsin and 1.5 mM CaCl_2_ in PBS). Dermis tissue was digested by incubation for 2 h at 37 °C with an enzyme cocktail made of 0.6 g/mL collagenase II (Biochrom, Berlin, Germany), 0.3 g/mL hyaluronidase (Sigma-Aldrich, Hamburg, Germany), and 1 μg/mL DNase (Roche Diagnostics, Berlin, Germany). Enzymes were stopped with RPMI-1640 cell culture medium (PAA, Heidelberg, Germany) containing 10% fetal calf serum (PAA, Heidelberg, Germany) and cells were harvested by repeated pipetting, filtering through a 70 µm cell strainer (FalconTM, Becton Dickinson, Heidelberg, Germany) and washing twice with PBS (PAA, Heidelberg, Germany). After centrifugation at 300× *g* for 10 min, cells were fixed with 4% paraformaldehyde (Sigma-Aldrich, Taufkirchen, Germany) and stored at 4 °C until analysis by flow cytometry (FACS Calibur, BD, Heidelberg, Germany). At least 20,000 events were collected in the selected gate. The software FCS Express (De Novo Software, Version 3.1, Glendale, CA, USA) was used for data analysis. Pictures of isolated cells were taken by means of a confocal laser microscope (LSM Exciter, Zeiss, Jena, Germany).

### 2.8. Isolation and Culture of T cells

T cells were isolated from dermis after digestion as described above. Dermis cell suspension was cultured overnight in RPMI 1640 medium (Gibco, Darmstadt, Germany) supplemented with 10% fetal calf serum, 100 µg/mL streptomycin, and 100 I.E./mL penicillin. Non-adherent cells were collected and red blood cells were lysed with 1% Triton X-100 in PBS for 10 min on ice. Cells were then washed with PBS and incubated with carboxyfluorescein succinimide ester (CFSE, CellTraceTM, Life Technologies, Darmstadt, Germany) for 20 min at room temperature. Cells were washed with 1% bovine serum albumin and re-suspended in supplemented RPMI 1640 medium. Cells were left untreated, incubated with IL-2 (2.5 µg/mL) only, or incubated with IL-2 and tacrolimus in 10% ethanol or tacrolimus-loaded NG-tPG nanogels (final tacrolimus concentration of 5 µg/mL). Cell proliferation was measured after 5 days by flow cytometry. T cells were gated in forward vs. side scatter dot plots and at least 10,000 events were collected.

### 2.9. Co-culture of Full-Thickness Human Skin and Jurkat Cells

After topical application of tacrolimus formulations, skin samples were transferred on 8 µm-pore inserts (Cell Culture Inserts, BD Falcon^™^, Corning, New York, NY, USA). These were placed in a 6-well plate with 2 mL supplemented RPMI 1640 medium and 10^6^ Jurkat cells (clone E6.1) per well. Jurkat cells had previously been stained with CFSE as described above. Skin was maintained at the air-liquid interface and co-cultured with Jurkat cells for 2 or 5 days (37 °C, 5% CO_2_). 500 µL of fresh medium were replaced every 2 days. Jurkat cells were collected and analyzed by flow cytometry.

## 3. Results and Discussion

### 3.1. Tacrolimus Penetration and Inflammatory Reaction in Ex Vivo Human Skin with Intact or Disrupted Barrier

**Figure 1:**
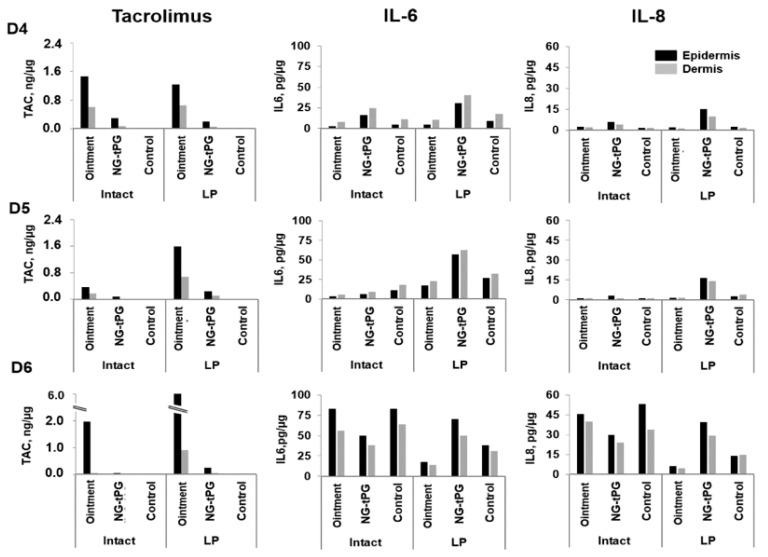
Tacrolimus skin penetration and expression of the inflammatory cytokines IL-6 and IL-8 after topical application of tacrolimus ointment or nanogel formulation (tacrolimus final dosage 5 µg/cm^2^) on breast skin from three different donors (D4–6). Skin barrier was left intact or treated with LP prior to the application of the test formulations. Control skin was treated with 0.9% NaCl solution. TAC: tacrolimus; LP: laser poration; NG-tPG: nanogels based on thermoresponsive polyglycerol.

**Figure 2:**
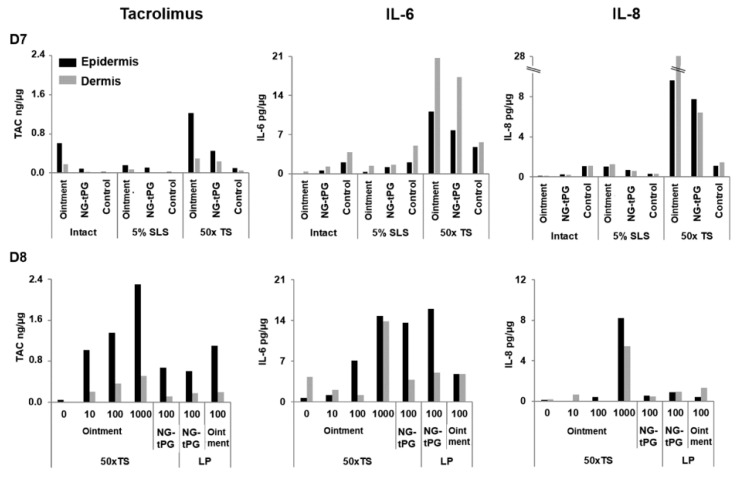
Effects of tape stripping (TS) on tacrolimus penetration and release of IL-6 and IL-8 in breast skin. The amounts of tacrolimus, IL-6, and IL-8 were measured in the epidermis and dermis of breast skin pre-treated to disrupt the skin barrier and incubated with the investigated tacrolimus formulations. (D7) Breast skin was treated with 50 × TS or with 5% SLS previous topical application of 0.1% tacrolimus ointment and tacrolimus-loaded NG-tPG (tacrolimus end concentration 5 µg/cm^2^) after 24 h. (D8) Tacrolimus penetration and cytokine release in breast skin pre-treated with 50 × TS after 10, 100, and 1000 min of incubation with tacrolimus ointment and comparison with skin pre-treated with 50 × TS or LP and incubation for 100 min with NG-tPG or ointment formulations. Control skin was treated with 0.9% NaCl solution. 50 × TS: tape stripping 50 times; TAC: tacrolimus; LP: laser poration; 5% SLS: 5% sodium lauryl sulfate; NG-tPG: nanogels based on thermoresponsive polyglycerol.

**Figure A1:**
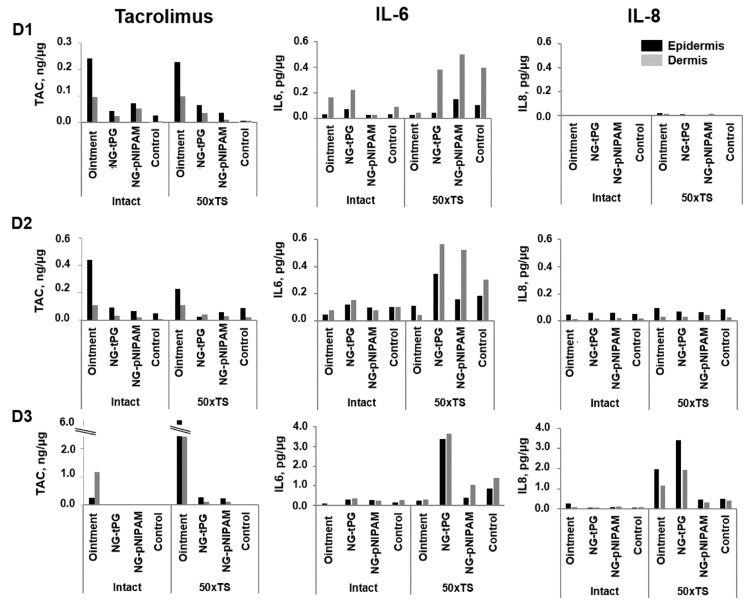
Tacrolimus penetration after topical application on abdominal skin with or without barrier disruption. Abdominal skin from three different donors (D1-3) was left intact or treated with tape stripping times (50 × TS). NG-pNIPAM (0.9 wt.% tacrolimus), NG-tPG (2.5 wt.% tacrolimus), and 0.1% tacrolimus ointment were applied for 24 h at a final tacrolimus concentration of 5 µg/cm^2^. Tacrolimus as well as the inflammatory markers IL-6 and IL-8 were detected in skin extracts. TAC: tacrolimus.

In order to compare the new nanogel-based tacrolimus formulations to the commercially available 0.1% tacrolimus ointment, we measured the amounts of penetrated drug and released inflammatory markers (IL-6 and IL-8) in ex vivo human skin. Different methods (TS, LP, and SLS) were applied to simulate a barrier dysfunction and improve drug penetration. Laser poration (at the used energy) induces a local removal of stratum corneum (micropores), whereas tape stripping reduces the thickness of the stratum corneum. Both methods result in a partial removal of both lipids and corneocytes [30,31]. On the other hand, the surfactant SLS acts predominantly on the lipid layers disrupting their structure [32]. Skin from eight donors and two different areas was used: abdomen (see Appendix A, Figure A1(D1–D3)) and breast (Figure 1(D4–D6), and Figure 2(D7,D8). After incubation, skin samples were processed as described in Material and Methods and the amounts of drug as well as cytokines were quantified by means of LC-MS/MS and ELISA, respectively. In general, the ointment formulation resulted in higher concentrations of penetrated tacrolimus. This better performance might be due to the permeabilizing effects of some of the excipients in the ointment formulation. However, it has to be considered that the detection of tacrolimus in epidermis extracts does not provide any spatially resolved information, i.e., the distribution of the drug between the SC and the viable epidermis remains unknown.

Low penetration of tacrolimus was measured in intact abdominal skin. Pretreatment with TS had no significant effects on drug penetration, independently on the tested formulation (Figure A1). Little effect was measured also with regard of the inflammatory markers IL-6 and IL-8. On the contrary, higher tacrolimus concentrations were detected in intact breast skin as compared to intact abdominal skin (Figure 1). In addition, slightly increased drug penetration and cytokine production were found after barrier disruption by LP.

These results suggest that breast skin is more permeable than abdominal skin, probably due to a thinner SC and a higher density of hair follicles, which have been shown to contribute to the skin permeation of topically applied substances [33,34,35]. The fact that skin from breast areas expressed also much more IL-6 and IL-8 than skin from abdominal region might be due to a higher number of immune active cells.

With regard to tacrolimus penetration in breast skin, TS pretreatment resulted in a marked increase of tacrolimus penetration for both ointment and nanogel formulations (Figure 2(D7,D8)), while application of 5% SLS, a standard procedure for chemical barrier disruption, had no effects. The results obtained after different time points (Figure 2(D8)) show that the penetration of tacrolimus in barrier disrupted skin treated with the ointment increased exponentially with time. On the contrary, the amount of drug penetrated in nanogel-treated samples after 100 min of incubation were similar to that measured after 24 h of incubation (Figure 2(D7)), suggesting a slower but constant delivery of drug by the nanogel formulations. Thus, the lower amounts of penetrated drug observed for the nanogel formulations after 24 h of incubation may also be a result of the slower drug delivery rate of the nanogel formulations.

When considering cytokine release, a clear increase of both inflammatory markers was observed in control skin after LP or TS especially in the experiments performed on breast skin (Figure 1 and Figure 2). An additional increase of IL-8 and IL-6 was registered in tacrolimus-treated barrier disrupted skin. This reaction might be attributed to activating or irritating effects of the nanogels themselves or of tacrolimus. While in previous experiments no toxicity was detected for the investigated nanogels [25,26], it is known that tacrolimus may have irritating effects [12]. Our findings point towards the fact that enhanced penetration, while beneficial for therapeutic effects, may pose new challenges with regard to tolerability, especially when the applied substances are capable of reaching cells of the viable epidermis that might be in an activated state as observed in inflammatory skin [36,37].

Overall, these results indicate that breast skin is more permeable to tacrolimus than abdominal skin, regardless of whether skin barrier was disrupted or not. After 24 h of incubation, higher amounts of tacrolimus were measured in ointment-treated samples. Nevertheless, biological effects, like the release of IL-6 and IL-8, were detected also in response to tacrolimus-loaded nanogel formulations.

### 3.2. Nanogel Skin Penetration and Cellular Uptake after Different Degrees of Barrier Disruption by TS

**Figure 3:**
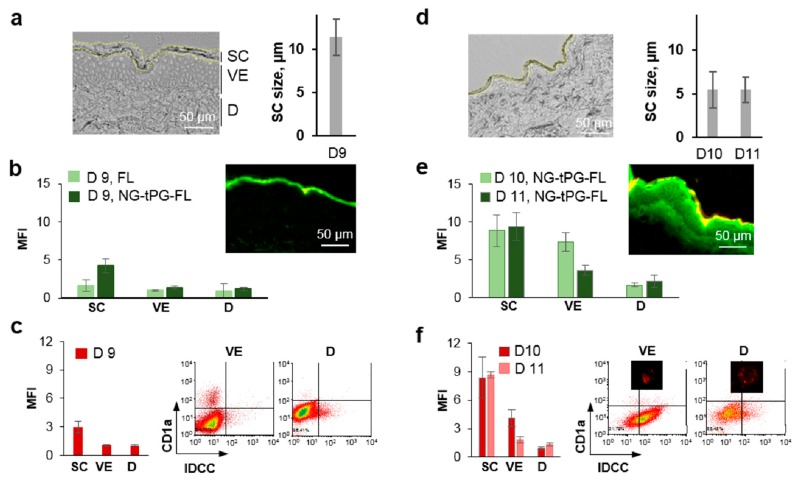
Skin penetration of topically applied nanogels and released dye depends on the degree of barrier disruption. Mild (**a**–**c**) and severe (**d**–**f**) skin barrier disruption was induced in breast skin by TS previous application of free fluorescein (green) or NG-tPG tagged with IDCC (red) and loaded with FL. After 16 h of incubation, skin was processed to prepare cryosections and isolate cells. (**a**,**d**) Representative transmission light microscopy images of skin sections showing the different degrees of barrier disruption and diagrams showing the average SC thickness; (**b**,**e**) analysis of FL penetration in SC, viable epidermis and dermis of skin sections by measurement of mean fluorescence intensity; (**c**,**f**) analysis of IDCC fluorescence on skin sections (diagrams) and in cells (flow cytometry, dot plots, and images of single cells) isolated from NG-tPG-treated skin and stained with anti-CD1a antibody (Langerhans cells). FL: fluorescein; SC: stratum corneum; VE: viable epidermis; D: dermis; MFI: mean fluorescence intensity; NG-tPG: nanogels based on thermoresponsive polyglycerol; IDCC: indodicarbocyanine.

The SC is a key barrier which hinders the penetration of topically applied compounds. Especially size is one of the major determinants of penetration. With regard to nanoparticulate drug delivery systems, deformability and elasticity further contribute to their penetration properties. Large amounts of carrier material typically remain on the skin surface or in superficial SC compartments. However, the question as to how deep single nanogel particles are capable to penetrate is important with regard to the likelihood of exposure of viable cells to nanogels. Thus, possible translocation of small amounts of nanogel particles to the viable skin layers after skin barrier disruption by TS was investigated using IDCC-tagged nanogels loaded with fluorescein (FL) (Figure 3).

The degree of skin barrier disruption was assessed by measuring the SC thickness in images of skin sections from different regions of the sample. In Figure 3a,d, representative images and the average of at least 15 measurements from three different donors (D9–11) are shown. After 50 TS, a mild skin barrier disruption was achieved for one donor (Figure 3a(D9)), whereas a more severe skin barrier disruption with a stronger reduction of SC thickness was obtained in skin from two other donors (Figure 3d, D10 and D11). Such a different extent of barrier disruption reflects the individual variability of SC thickness and strength. The spatially resolved skin penetration of nanogels (IDCC, red fluorescence) and delivered model dye (fluorescein, FL, green fluorescence) served to clarify the drug delivery mechanism of nanogels. In the representative fluorescence images (Figure 3b,e), it is to recognize that the two fluorophores co-localized in the SC, which resulted in yellow fluorescence, whereas the released FL penetrated deeper in the viable epidermis, especially in the skin with severe barrier disruption. To quantify the extent of FL penetration, the mean fluorescence intensity (MFI) in the green channel was calculated for different skin areas of at least 20 skin sections per sample. For D9 (Figure 3b), a higher fluorescence signal was detected in the SC for the tNG sample as compared to free FL, confirming the ability of NG-tPG to create a depot in the outermost layer of the epidermis [22]. In skin from donors 10 and 11, where TS procedure had induced a severe skin barrier disruption, a better skin penetration of the released dye was observed not only in the SC but also in the viable epidermis and dermis (Figure 3e). The analysis of NG-tPG penetration (IDCC signal) by fluorescence microscopy showed penetration only in the SC for donor 9 and also in the viable epidermis for donors 10 and 11 (Figure 3c,f). To detect possible cellular association with penetrated nanogels, part of the treated skin was processed to isolate cells and analyze them by flow cytometry and confocal fluorescence microscopy. While after mild skin disruption (D9) only a small percent of cells had high fluorescent signal, over 50% of epidermis cells and 10% of dermis cells were associated with nanogels after severe barrier perturbation (D10 and D11). Especially in the two donors with severe skin barrier disruption, NG-tPG were found to be associated also with Langerhans cells.

These results clearly show that, when skin barrier is compromised, nanogels can penetrate to viable skin layers, be taken-up by epidermal and dermal cells and that uptake in dendritic cells is favored in pro-inflammatory environment. This observation may be of special advantage for the treatment of inflammatory skin diseases where, besides T cells, dendritic cells are key targets of therapeutics like tacrolimus. [5,6].

### 3.3. Effects of Tacrolimus Nanogels and Ointment on T Cells and Skin/Jurkat Cell Co-Cultures.

**Figure 4:**
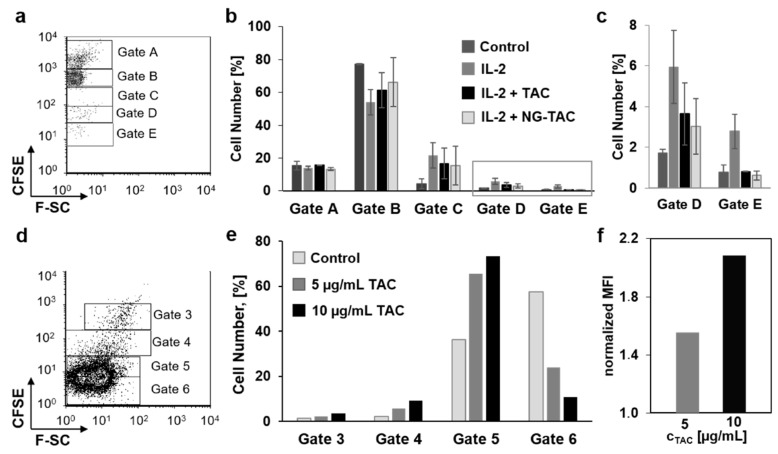
Effects of tacrolimus in solution or formulated in nanogels on Jurkat and T-cell proliferation in vitro. (**a**–**c**) Preliminary experiments on isolated dermal T-cells stimulated with IL-2 showed the inhibitory effects of tacrolimus (5 µg/mL) both in solution and in nanogels. Different cell populations were detected by flow cytometry (**a**) and the percentage of cells in each gate at day 5 of culture are plotted (**b**). Values in gates D and E are reported in (**c**) using a different axis scale. (**d**–**f**) Effects of tacrolimus were also detected in Jurkat cells after incubation with 5 and 10 µg/mL tacrolimus solution for 4 days. Cells were gated according to fluorescence intensity (**d**). The percentages of cells in each gate (e) as well as the normalized mean fluorescence intensity of all cells (**f**) showed a decrease of proliferation after treatment with tacrolimus. MFI: mean fluorescence intensity.

As outlined in the previous sections, assessment of a potential benefit of enhanced tacrolimus penetration on the cellular level is limited by its irritative effects on keratinocytes and fibroblasts. Given that immune cells are main therapeutic targets, we created an experimental set-up of skin tissue/T cell co-cultures to measure the effects of penetrated tacrolimus on T cell proliferation [3,10]. First, tacrolimus anti-proliferative effects was measured in vitro on T cells isolated from human excised skin and stimulated with recombinant human IL-2 (Figure 4a–c).

Approximately 20% of cells were stimulated to proliferate after addition of IL-2. Both tacrolimus in solution and loaded on NG-tPG reproducibly reduced the percentage of proliferating cells (Figure 4b,c). Tacrolimus was also reported to exert anti-proliferative effects on T cells and Jurkat cells via induction of apoptosis [7,8]. Accordingly, when Jurkat cells were incubated with tacrolimus, a concentration dependent anti-proliferative effect could be measured (Figure 4d–f). These results show the ability of nanogels to deliver tacrolimus to T cells in vitro and induce anti-proliferative effects in a degree similar to that of the free drug. In addition, it was shown that the pro-apoptotic effects of tacrolimus on Jurkat cells were measurable by CFSE assay (Figure 4e,f).

**Figure 5:**
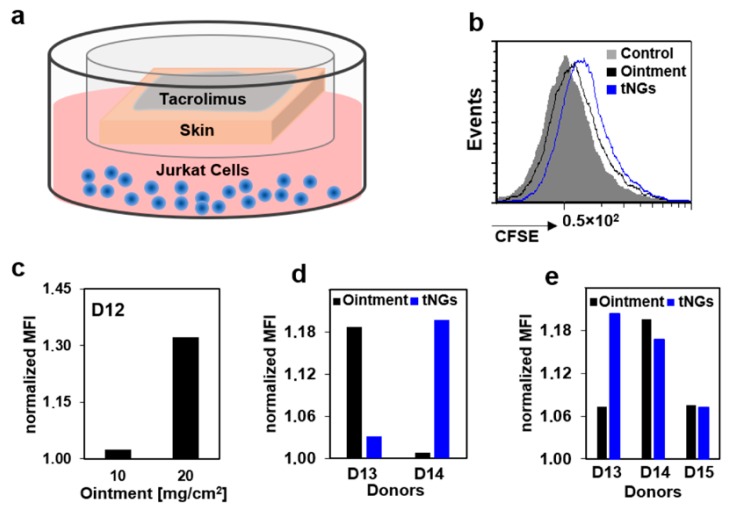
Penetration of tacrolimus across full thickness skin and inhibition of Jurkat cell proliferation. (**a**) Typical experimental procedure. Tacrolimus ointment or nanogels were applied topically on ex vivo skin with disrupted barrier (tape stripping 50 times) that was co-cultured with CFSE-labelled Jurkat cells in a trans-well set up. (**b**) Flow cytometry histogram of cells after treatment with tacrolimus ointment. (**c**) Normalized mean fluorescence intensity of cells cocultured with skin treated with 10 and 20 mg/cm^2^ of ointment. (**d**,**e**) Normalized mean fluorescence intensity of Jurkat cells co-cultured for 2 (**d**) or 5 days (**e**) with skin treated with 5 µg/cm^2^ of tacrolimus formulated as ointment or nanogel. Both ointment and tacrolimus-loaded NG showed inhibitory effects on Jurkat cell proliferation after topical application on co-cultured ex vivo skin. TAC: tacrolimus; CFSE: carboxyfluorescein succinimide ester; MFI: mean fluorescence intensity; tNGs: thermoresponsive nanogels.

As next step, we cultured ex vivo skin at the air/liquid interface with Jurkat cells using a trans-well set-up (Figure 5a).

We hypothesized that, when tacrolimus is applied topically on skin, the penetrated drug would reach the medium compartment and affect the proliferation of Jurkat cells. In fact, all treated samples had higher MFI than the controls, as shown in the representative histogram in Figure 5b, confirming the anti-proliferative effects of the penetrated drug. A concentration-dependent effect was visible when Jurkat cells were co-cultured for 4 days with skin topically treated with 10 and 20 mg/cm^2^ of 0.1% tacrolimus ointment, corresponding to 10 and 20 µg/cm^2^ of tacrolimus, respectively (Figure 5c). When the ointment was compared to the NG-tPG tacrolimus formulation using a final tacrolimus concentration of 5 µg/cm^2^ (Figure 5d,e), a clear reduction of proliferation was visible at day 5 (Figure 5e). Interestingly, at day 5, ointment and nanogels had similar effects for donors D14 and D15, while for donor D13 nanogels had even a higher anti-proliferative activity than the ointment. Thus, even if in the 24 h penetration experiments, low tacrolimus amounts were measured in the skin treated with nanogels, after longer incubation time in the skin/cell co-culture set up, nanogel formulations could exert anti-proliferative effects comparable to that of tacrolimus ointment.

## 4. Conclusions

This In this work, we show that tacrolimus formulated as ointment or nanogel suspension penetrates skin with different efficiency in dependence on SC thickness and integrity. Irritation effects of tacrolimus ointment and nanogel formulations, reflected by the released inflammatory cytokines IL-6 and IL-8, were more pronounced in barrier-disrupted and immuno-activated skin. Extensive barrier disruption by mechanical removal of SC resulted also in enhanced penetration of topically applied materials, including deformable macromolecules like nanogels, with consequent interaction and uptake by skin immune active cells. These results support the key role of the SC as barrier for drug and nanocarrier penetration. Our results further demonstrate the critical balance of penetration enhancement and potential increase of side effects. Slow release systems as observed herein already addresses this aspect. The slow drug release and delivery combined with SC hydration and special interaction with skin cells in inflamed skin may explain the good performance of nanogels with respect to tacrolimus ointment. Thus, we conclude that tNGs represent an attractive alternative water-based formulation for the topical delivery of high molecular weight, poorly water-soluble drugs like tacrolimus.

## Figures and Tables

**Table 1:** Properties and drug loading of the investigated nanogels.

Nanogel	Size (PDI) ^a^	Cloud Point Temperature ^b^	Tacrolimus Loading ^c^	ζ Potential [mV] ^d^
NG-pNIPAM	110.5 nm (0.194)	34.6 °C	0.9 wt.%	−1.07
NG-tPG	132.9 nm (0.073)	28.9 °C	2.5 wt.%	0.332

^a^ Size and polydispersity index (PDI) by dynamic light scattering (DLS) in water at 25 °C. Measurements were performed in triplicates; intensity average mean value presented; ^b^ Cloud point temperature determined as the temperature at 50% transmittance by UV-Vis (λ = 500 nm); ^c^ Refer to Gerecke, et al., *Nanotoxicology* **2017**, *11*, 267–277 [26] for detailed information; ^d^ ζ potential determined by Zeta-sizer in phosphate buffer. Measurements were performed in triplicates; intensity average mean value presented. NG-pNIPAM: poly(*N*-isopropylacrylamide-based nanogels; NG-tPG: poly(glycidyl methyl ether-co-ethyl glycidyl ether)-based nanogels.

## Appendix A

Tacrolimus penetration and inflammatory reaction in abdominal skin with or without barrier disruption. Tape stripping had no significant effects on drug penetration in abdominal skin. Only in skin from one donor and only for the ointment a high amount of tacrolimus, approximately 6 ng per µg of total extracted proteins, was detected after tape stripping (Figure A1, Donor 3). Little effect was measured also with regard of the inflammatory markers IL-6 and IL-8, with values around 0.2 pg per µg of total proteins and maximal of 4 pg/µg for NG-tPG in donor 3. While a moderate increase of IL-6 could be observed in tape-stripped skin controls, almost no IL-8 increase was measured in controls after barrier disruption, indicating that IL-6 might be a more sensitive marker for measuring the effect of mechanical damage to the SC. A moderate increase of IL-6 with respect to controls was observed only in the samples treated with the nanogel formulations, whereas a slight decrease of IL-6 with respect to control was measured in skin treated with 0.1% tacrolimus ointment.

## References

[1] Liu J., Farmer J.D., Lane W.S., Friedman J., Weissman I., Schreiber S.L. (1991). Calcineurin is a common target of cyclophilin-cyclosporin A and FKBP-FK506 complexes. Cell.

[2] McCaffrey P.G., Perrino B.A., Soderling T.R., Rao A. (1993). NF-ATp, a T lymphocyte DNA-binding protein that is a target for calcineurin and immunosuppressive drugs. J. Biol. Chem..

[3] Brazelton T.R. (1996). Molecular mechanisms of action of new xenobiotic immunosuppressive drugs: Tacrolimus (FK506), sirolimus (rapamycin), mycophenolate mofetil and leflunomide. Curr. Opin. Immunol..

[4] De Paulis A., Stellato C., Cirillo R., Ciccarelli A., Oriente A., Marone G. (1992). Anti-inflammatory effect of FK-506 on human skin mast cells. J. Investig. Dermatol..

[5] Panhans-Groß A., Novak N., Kraft S., Bieber T. (2001). Human epidermal Langerhans’ cells are targets for the immunosuppressive macrolide tacrolimus (FK506). J. Aller. Clin. Immunol..

[6] Wollenberg A., Sharma S., von Bubnoff D., Geiger E., Haberstok J., Bieber T. (2001). Topical tacrolimus (FK506) leads to profound phenotypic and functional alterations of epidermal antigen-presenting dendritic cells in atopic dermatitis. J. Aller. Clin. Immunol..

[7] Hashimoto Y., Matsuoka N., Kawakami A., Tsuboi M., Nakashima T., Eguchi K., Tomioka T., Kanematsu T. (2001). Novel immunosuppressive effect of FK506 by augmentation of T cell apoptosis. Clin. Exper. Immunol..

[8] Chung Y., Chung M., Choi S., Choi S., Choi S., Chung S. (2018). Tacrolimus-Induced Apoptosis is Mediated by Endoplasmic Reticulum–derived Calcium-dependent Caspases-3,-12 in Jurkat Cells. Transplant. Proc..

[9] Matsuda S., Shibasaki F., Takehana K., Mori H., Nishida E., Koyasu S. (2000). Two distinct action mechanisms of immunophilin–ligand complexes for the blockade of T-cell activation. EMBO Rep..

[10] Soter N.A., Fleischer A.B., Webster G.F., Monroe E., Lawrence I., Group T.O.S. (2001). Tacrolimus ointment for the treatment of atopic dermatitis in adult patients: Part II, safety. J. Am. Acad. Dermatol..

[11] Lan C.-C.E., Yu H.-S., Huang S.-M., Wu C.-S., Chen G.-S. (2007). FK506 induces interleukin-6 secretion from UVB irradiated cultured human keratinocytes via p38 mitogen-activated protein kinase pathway: Implication on mechanisms of tacrolimus-induced skin irritation. J. Dermatol. Sci..

[12] Muraoka K., Fujimoto K., Sun X., Yoshioka K., Shimizu K., Yagi M., Bose H., Miyazaki I., Yamamoto K. (1996). Immunosuppressant FK506 induces interleukin-6 production through the activation of transcription factor nuclear factor (NF)-kappa (B). Implications for FK506 nephropathy. J. Clin. Investig..

[13] Shokrollahi K. (2017). Paraffin-Based Ointments and Fire Hazard: Understanding the Problem, Navigating the Media and Currently Available Downloadable Patient Information.

[14] Pople P.V., Singh K.K. (2013). Development and evaluation of colloidal modified nanolipid carrier: Application to topical delivery of tacrolimus, Part II–In vivo assessment, drug targeting, efficacy, and safety in treatment for atopic dermatitis. Eur. J. Pharm. Biopharm..

[15] Erdogan M., Wright J., McAlister V. (2002). Liposomal tacrolimus lotion as a novel topical agent for treatment of immune-mediated skin disorders: Experimental studies in a murine model. Br. J. Dermatol..

[16] Lapteva M., Mondon K., Möller M., Gurny R., Kalia Y.N. (2014). Polymeric micelle nanocarriers for the cutaneous delivery of tacrolimus: A targeted approach for the treatment of psoriasis. Mol. Pharm..

[17] Li G., Fan Y., Fan C., Li X., Wang X., Li M., Liu Y. (2012). Tacrolimus-loaded ethosomes: Physicochemical characterization and in vivo evaluation. Eur. J. Pharm. Biopharm..

[18] Yamamoto K., Klossek A., Fuchs K., Watts B., Raabe J., Flesch R., Rancan F., Pischon H., Radbruch M., Gruber A. (2019). Soft X-ray microscopy for probing of topical tacrolimus delivery via micelles. Eur. J. Pharm. Biopharm..

[19] Lei W., Yu C., Lin H., Zhou X. (2013). Development of tacrolimus-loaded transfersomes for deeper skin penetration enhancement and therapeutic effect improvement in vivo. Asian J. Pharm. Sci..

[20] Cuggino J.C., Blanco E.R.O., Gugliotta L.M., Igarzabal C.I.A., Calderón M. (2019). Crossing biological barriers with nanogels to improve drug delivery performance. J. Control. Release.

[21] Rancan F., Asadian-Birjand M., Dogan S., Graf C., Cuellar L., Lommatzsch S., Blume-Peytavi U., Calderón M., Vogt A. (2016). Effects of thermoresponsivity and softness on skin penetration and cellular uptake of polyglycerol-based nanogels. J. Control. Release.

[22] Giulbudagian M., Rancan F., Klossek A., Yamamoto K., Jurisch J., Neto V.C., Schrade P., Bachmann S., Rühl E., Blume-Peytavi U. (2016). Correlation between the chemical composition of thermoresponsive nanogels and their interaction with the skin barrier. J. Control. Release.

[23] Rancan F., Giulbudagian M., Jurisch J., Blume-Peytavi U., Calderon M., Vogt A. (2017). Drug delivery across intact and disrupted skin barrier: Identification of cell populations interacting with penetrated thermoresponsive nanogels. Eur. J. Pharm. Biopharm..

[24] Giulbudagian M., Yealland G., Hönzke S., Edlich A., Geisendörfer B., Kleuser B., Hedtrich S., Calderón M. (2018). Breaking the Barrier-Potent Anti-Inflammatory Activity following Efficient Topical Delivery of Etanercept using Thermoresponsive Nanogels. Theranostics.

[25] Edlich A., Gerecke C., Giulbudagian M., Neumann F., Hedtrich S., Schäfer-Korting M., Ma N., Calderon M., Kleuser B. (2017). Specific uptake mechanisms of well-tolerated thermoresponsive polyglycerol-based nanogels in antigen-presenting cells of the skin. Eur. J. Pharm. Biopharm..

[26] Gerecke C., Edlich A., Giulbudagian M., Schumacher F., Zhang N., Said A., Yealland G., Lohan S.B., Neumann F., Meinke M.C. (2017). Biocompatibility and characterization of polyglycerol-based thermoresponsive nanogels designed as novel drug-delivery systems and their intracellular localization in keratinocytes. Nanotoxicology.

[27] Dzhonova D., Olariu R., Leckenby J., Dhayani A., Vemula P.K., Prost J.-C., Banz Y., Taddeo A., Rieben R. (2018). Local release of tacrolimus from hydrogel-based drug delivery system is controlled by inflammatory enzymes in vivo and can be monitored non-invasively using in vivo imaging. PLoS ONE.

[28] Giulbudagian M., Asadian-Birjand M., Steinhilber D., Achazi K., Molina M., Calderón M. (2014). Fabrication of thermoresponsive nanogels by thermo-nanoprecipitation and in situ encapsulation of bioactives. Polym. Chem..

[29] Koster R.A., Alffenaar J.-W.C., Greijdanus B., Uges D.R. (2013). Fast LC-MS/MS analysis of tacrolimus, sirolimus, everolimus and cyclosporin A in dried blood spots and the influence of the hematocrit and immunosuppressant concentration on recovery. Talanta.

[30] Döge N., Avetisyan A., Hadam S., Pfannes E.K.B., Rancan F., Blume-Peytavi U., Vogt A. (2017). Assessment of skin barrier function and biochemical changes of ex vivo human skin in response to physical and chemical barrier disruption. Eur. J. Pharm. Biopharm..

[31] Bachhav Y., Summer S., Heinrich A., Bragagna T., Böhler C., Kalia Y. (2010). Effect of controlled laser microporation on drug transport kinetics into and across the skin. J. Control. Release.

[32] Yanase K., Hatta I. (2018). Disruption of human stratum corneum lipid structure by sodium dodecyl sulphate. Inter. J. Cosmet. Sci..

[33] Otberg N., Patzelt A., Rasulev U., Hagemeister T., Linscheid M., Sinkgraven R., Sterry W., Lademann J. (2008). The role of hair follicles in the percutaneous absorption of caffeine. Br. J. Clin. Pharm..

[34] Mohd F., Todo H., Yoshimoto M., Yusuf E., Sugibayashi K. (2016). Contribution of the hair follicular pathway to Total skin permeation of topically applied and exposed chemicals. Pharmaceutics.

[35] Tampucci S., Burgalassi S., Chetoni P., Lenzi C., Pirone A., Mailland F., Caserini M., Monti D. (2014). Topical formulations containing finasteride. Part II: Determination of finasteride penetration into hair follicles using the differential stripping technique. J. Pharm. Sci..

[36] Rancan F., Amselgruber S., Hadam S., Munier S., Pavot V., Verrier B., Hackbarth S., Combadiere B., Blume-Peytavi U., Vogt A. (2014). Particle-based transcutaneous administration of HIV-1 p24 protein to human skin explants and targeting of epidermal antigen presenting cells. J. Control. Release.

[37] De Benedetto A., Kubo A., Beck L.A. (2012). Skin barrier disruption: A requirement for allergen sensitization?. J. Investig. Dermatol..

